# Capsule-deficient group A *Streptococcus* evades autophagy-mediated killing in macrophages

**DOI:** 10.1128/mbio.00771-24

**Published:** 2024-05-31

**Authors:** Yong-An Shi, Shiou-Ling Lu, Takeshi Noda, Cheng-Hsun Chiu, Chuan Chiang-Ni

**Affiliations:** 1 Graduate Institute of Biomedical Sciences, College of Medicine, Chang Gung University, Taoyuan, Taiwan; 2 Department of Microbiology and Immunology, College of Medicine, Chang Gung University, Taoyuan, Taiwan; 3 Center for Frontier Oral Science, Graduate School of Dentistry, Osaka University, Osaka, Japan; 4 Graduate School of Frontier Biosciences, Osaka University, Osaka, Japan; 5 Molecular Infectious Disease Research Center, Chang Gung Memorial Hospital at Linkou, Taoyuan, Taiwan; The Ohio State University, Columbus, Ohio, USA; Kokuritsu Kansensho Kenkyujo, Shinjyuku-ku, Tokyo, Japan

**Keywords:** hyaluronic acid capsule, autophagy, macrophages, group A *Streptococcus*

## Abstract

The hyaluronic acid capsule is crucial in protecting group A *Streptococcus* (GAS) against phagocytic killing. However, there have been reported outbreaks caused by capsule-deficient GAS strains, and the mechanisms underlying their evasion of immune clearance remain unclear. This study demonstrated that the capsule-deficient mutant [Cap(−)] of the *emm1* strain increased survival within phagocytic cells compared to the wild-type strain [Cap(+)]. Although both Cap(+) and Cap(–) strains exhibited similar abilities to disrupt the phagosome, only the Cap(+) strain was colocalized with lysosomes and acidified compartments in phagocytic cells, indicating its susceptibility to autophagosome elimination. In contrast, the Cap(–) mutant evaded the recognition of galectin-8 and ubiquitin, impairing selective autophagy-mediated elimination. These findings suggest that a deficiency in the capsule could impair the intracellular elimination of GAS in macrophages, revealing previously unknown aspects of the host’s recognition of the GAS capsule in macrophages.

**IMPORTANCE:**

Group A *Streptococcus* (GAS) is a Gram-positive bacterium that causes diseases ranging from mild pharyngitis to severe necrotizing fasciitis. Phagocytic cells serve as the primary defense against bacterial infections, exhibiting remarkable efficiency in eliminating intracellular pathogens. The hyaluronic acid capsule is a critical virulence factor that contributes to the resistance of phagocytosis in GAS. Nevertheless, the outbreaks caused by GAS strains that lack the hyaluronic acid capsule have been reported, and the selective advantage of capsule-deficient strains during infection is not fully understood. This study showed that the autophagic adaptor proteins recognize the capsulated GAS strain but not the capsule-deficient mutant, indicating that the hyaluronic acid capsule could be the autophagic target in macrophages. These findings imply that the hyaluronic acid capsule of GAS actually enhances its elimination within phagocytic cells, subverting the understanding of the capsule in GAS pathogenesis.

## INTRODUCTION

*Streptococcus pyogenes* (group A *Streptococcus*, GAS) is a Gram-positive bacterium that causes a wide variety of diseases, including scarlet fever, pharyngitis, impetigo, cellulitis, necrotizing fasciitis, toxic shock syndrome, as well as the sequelae, including rheumatic fever and acute post-streptococcal glomerulonephritis (1, 2). Hyaluronic acid is one of the components of GAS capsular polysaccharide (3). The *has* operon, contains *hasA*, *hasB*, and *hasC* genes, is responsible for producing the hyaluronic acid capsule. Studies demonstrated that the virulence of mucoid or highly encapsulated strains in mouse infection models was decreased after the hyaluronidase treatment *in vitro* (4–6), indicating that the hyaluronic acid capsule could protect GAS from phagocytosis. Furthermore, the hyaluronic acid capsule triggers cytoskeleton rearrangement by binding to CD44 on epithelial cells, resulting in the paracellular translocation of GAS from the epithelial surface into deep tissues (7). These results suggest the hyaluronic acid capsule is the critical virulence factor for GAS infection.

Strikingly, the emergence of capsule-deficient *emm89* GAS has been reported in invasive infections worldwide (8). Moreover, the GAS infections caused by capsule-deficient isolates increased at the pediatric hospital in Texas, USA (9). Zhu et al. (8) showed that the acapsular *emm89* isolates, which have the sequence variant in the *ifs-nga-slo* operon (P*nga*3), express a high level of SLO and NADase to compensate for the negative effect of the capsule deficiency. Interestingly, the *emm89* acapsular strain is equally virulent to the heavily encapsulated strain but is more virulent than its poorly encapsulated mutant in a mouse necrotizing fasciitis model (8). These findings not only indicate that the hyaluronic acid capsule production is dispensable for full virulence of *emm89* strains but also suggest the low level of hyaluronic acid capsule production would diminish their virulence in the mouse infection model.

Phagocytic cells play critical roles in the host’s innate immune defense against bacterial infection. After phagocytosis, the phagosome matures and fuses with lysosomes to clear the bacteria. Nevertheless, certain bacteria, such as *Listeria monocytogenes* and GAS, can rupture the phagosome and escape into the cytoplasm (10, 11). Subsequently, the cytosolic bacteria would be recognized by the specific autophagic adaptor proteins (e.g., galectin and ubiquitin), enclosed within autophagosomes, and eliminated after lysosome fusion (12–14). In contrast, GAS can evade autophagy-mediated killing and survive in the intracellular niche for several days (15, 16). This is achieved through the synergistic effects of GAS virulence factors. The secretion of the SpeB cysteine protease effectively degrades autophagy adaptors, thereby impeding autophagosome formation (17). SLO prevents the maturation of GAS-containing autophagosomes in keratinocytes (18). Furthermore, O’Neill et al. (19) demonstrated that in the absence of SLO, the cytotoxicity, vacuole rupture, and bacterial replication are significantly reduced compared to the wild-type strain, suggesting SLO is a crucial contributor to GAS to survive and replicate in macrophages (19, 20).

The hyaluronic acid capsule of GAS has been considered a key virulence factor for GAS survival from phagocytic killing since 1959 (5). The outbreaks of acapsular GAS strains were reported; however, the selective advantage/mechanism of acapsular mutants, particularly in resistance to phagocytic killing, is not understood. In this study, we investigated the roles of the hyaluronic acid capsule in GAS survival in the intracellular niche of phagocytic cells. Results showed that, after escape from the phagosome, the capsule-deficient strain was ineffectively recognized by galectin-8 and ubiquitin, resulting in better survival and replication activities in macrophages than the wild-type strain. These results uncovered the previously unknown interactions between the host defense system and the hyaluronic acid capsule of GAS.

## RESULTS

### Hyaluronic acid capsule-deficient GAS survives phagocytic killing

**Fig 1:**
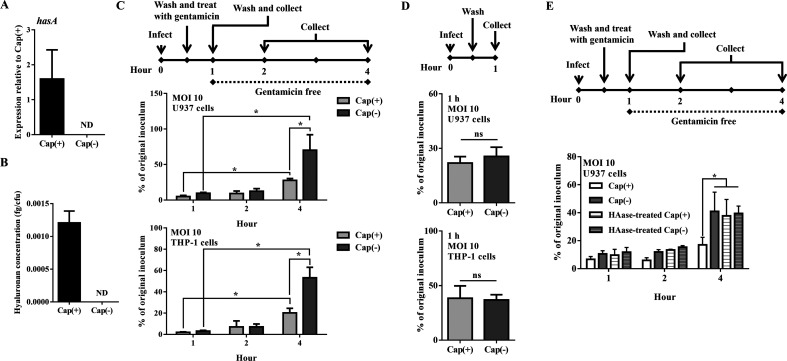
The Cap(–) mutant was more resistant to phagocytic killing than the Cap(+) strain. (**A**) The expression of *hasA* in the capsulated [Cap(+)] and capsule-deficient [Cap(–)] strains in the late exponential phase of growth [the optical density at 600 nm (OD_600_) reached 1.0]. RNAs of these strains were extracted, and the expression of *hasA* was analyzed by the quantitative RT-PCR. The expression of *hasA* was normalized to the expression of *gyrA* for both the Cap(+) and Cap(−) strains. (**B**) The production of the hyaluronic acid capsule in the Cap(+) and Cap(–) strains. The hyaluronic acid capsule of these strains was extracted and evaluated by the Hyaluronan Quantikine ELISA Kit. (**C**) The number of viable Cap(+) and Cap(–) strains in PMA-activated U937 and THP-1 cells. GAS strains infected PMA-activated U937 or THP-1 cells at an MOI of 10. After incubation for 30 min, the cells were treated with gentamicin (100 µg/mL) for an additional 30 min. Afterward, the medium was replaced by the gentamicin-free medium. The infected cells and culture media were collected to determine the number of surviving bacteria by plating assay. Data were analyzed by two-way ANOVA. (**D**) The number of cell-associated Cap(+) and Cap(–) strains after 1 h of infection at an MOI of 10 in PMA-activated U937 or THP-1 cells. Data were analyzed by Student’s *t*-test. (**E**) GAS strains were treated with hyaluronidase (1 µg/mL) for 30 min prior to infection. The number of Cap(+) strain, Cap(–) mutant, and the hyaluronidase-treated bacteria in the PMA-activated U937 cells. The infected cells and culture media were collected to determine the number of survival bacteria by plating assay. Data were analyzed by two-way ANOVA. ND, not detectable. ns, not significant. The mean ± SD of three independent experiments is shown. *P < 0.05.

To elucidate the role of the hyaluronic acid capsule in GAS infection, the ability of the wild-type strain [Cap(+)] and the capsule-deficient *hasA* mutant [Cap(–)] resistance to phagocytic killing was compared. The deletion of *hasA* and the deficient of hyaluronic acid capsule production in the Cap(–) mutant were verified by RT-qPCR and ELISA methods (Fig. 1A and B). In the phorbol myristate acetate (PMA)-activated U937 and THP-1 cell infection models, infected cells and culture media were collected to determine the number of surviving bacteria (Fig. 1C). The results showed that, in both U937 and THP-1 cells, the numbers of viable GAS were similar between Cap(+) and Cap(–) strains after 1–2 h of infection (Fig. 1C). However, after 4 h of infection, nearly 2.5-fold more viable bacteria can be recovered from Cap(–) mutant-infected cells than from Cap(+) strain-infected cells (Fig. 1C). The number of cell-associated Cap(+) and Cap(–) strains in the PMA-activated U937 and THP-1 cells was similar (Fig. 1D), indicating that the difference in survival activity between Cap(+) and Cap(–) strains was not due to the ability of GAS to adhere to phagocytes. Additionally, the cytotoxicity of Cap(+) and Cap(−) strains during 1–4 h of infection in the U937 cell infection model was compared by the LDH release assay, and no significant difference was observed (data not shown). Furthermore, the Cap(+) and Cap(–) strains were treated with hyaluronidase prior to infection to evaluate the role of the hyaluronic acid capsules on bacterial survival in phagocytic cells. The number of viable hyaluronidase-treated Cap(+) strain was increased compared with the cells infected by the untreated Cap(+) strain (Fig. 1E). No difference was found in cells infected by hyaluronidase-treated and untreated Cap(–) mutant (Fig. 1E). These results suggested that the acapsular strain would be more resistant to intracellular killing than the capsulated GAS strain.

### Hyaluronic acid capsule has no effect on GAS growth in the extracellular niche of phagocytes

The results from Fig. 1 suggested that the acapsular strain would be more resistant to phagocytic killing than the capsulated strain. To exclude the possibility that this difference is due to the increased growth activity of Cap(–) mutant outside macrophages, the number of extracellular Cap(+) and Cap(–) strains after 1–4 h of infection was compared. The PMA-activated U937 cells were treated with cytochalasin D to inhibit phagocytosis, and the number of viable extracellular bacteria was determined (Fig. S1A). Results showed the number of extracellular Cap(+) and Cap(–) strains was similar (Fig. S1B). Only a few intracellular bacteria can be recovered from the cytochalasin D-treated cells after gentamicin treatments (Fig. S1C), indicating that cytochalasin D effectively inhibited phagocytosis. In the absence of gentamicin treatments, the number of viable Cap(+) and Cap(–) strains was similar after 1–4 h of infection, indicating that Cap(+) and Cap(–) strains had similar growth activity in the extracellular niche of phagocytic cells.

### Hyaluronic acid capsule promotes intracellular killing in phagocytes

**Fig 2:**
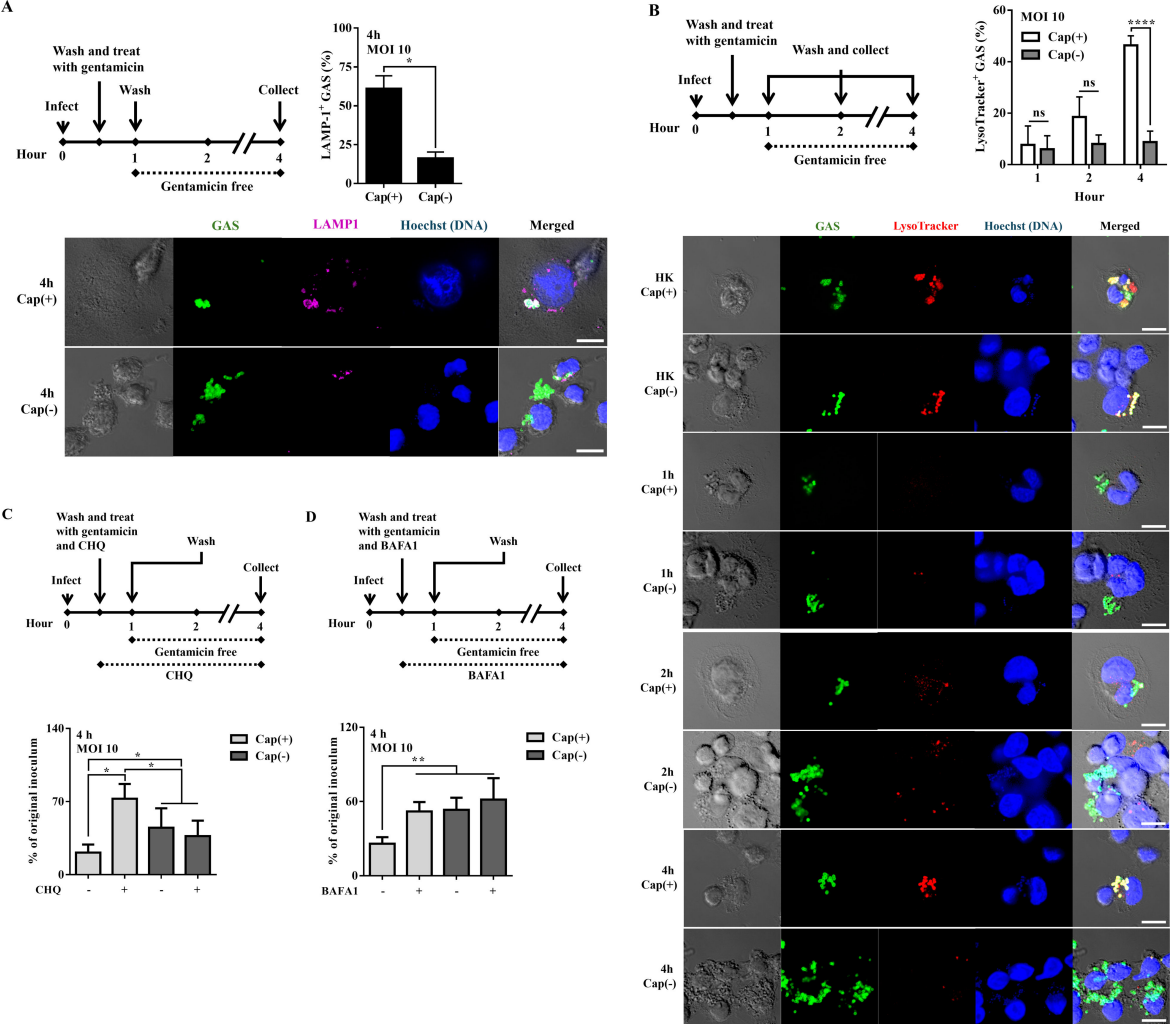
The Cap(+) strain but not Cap(–) mutant was effectively colocalized with mature phagosomes or autophagosomes. (**A**) The percentage of LAMP-1-colocalized Cap(+) and Cap(–) strains in PMA-activated U937 cells after 4 h of infection. GAS strains infected PMA-activated U937 cells at an MOI of 10. After incubation for 30 min, cells were incubated with gentamicin (100 µg/mL) for an additional 30 min. Afterward, the medium was replaced by the gentamicin-free medium. GAS and LAMP-1 were labeled with anti-GAS antibody (green) and anti-LAMP-1 antibody (cyan). DNA was stained with Hoechst 33342 (blue). Data were analyzed by Student’s *t*-test. ns, not significant. (**B**) The level of lysosome-associated Cap(+) and Cap(–) strains in PMA-activated U937 cells. GAS strains infected PMA-activated U937 cells at an MOI of 10. After incubation for 30 min, cells were incubated with gentamicin (100 µg/mL) for an additional 30 min. Afterward, the medium was replaced by the gentamicin-free medium. Representative images of PMA-activated U937 cells infected with Cap(+) strains, Cap(–) mutant, and the heat-killed (HK) bacteria (after 1 h of infection) by confocal microscopy are shown. GAS-infected U937 cells were stained with LysoTracker dye (Red) for 30 min prior to each time point. GAS was labeled with an anti-GAS antibody (green). DNA was stained with Hoechst 33342 (blue). Data were analyzed by two-way ANOVA. Percentages of LAMP-1-positive (**A**) and LysoTracker-positive GAS (**B**) were determined relative to total intracellular GAS. At least 50 infected cells were evaluated in three independent experiments (mean ± SD). (**C** and **D**) The number of surviving Cap(+) and Cap(–) strains in phagocytic cells treated with the lysosomal inhibitors (CHQ and BAFA1) after 4 h of infection. GAS strains infected PMA-activated U937 cells at an MOI of 10. After incubation for 30 min, cells were incubated with gentamicin (100 µg/mL), CHQ (50 µM), and BAFA1 (100 nM) for an additional 30 min. Afterward, the medium was replaced by the gentamicin-free medium that contained CHQ or BAFA1. After 4 h of infection, the infected cells and culture media were collected to determine the number of surviving bacteria by plating assay. Data were analyzed by one-way ANOVA. The mean ± SD of three independent experiments is shown. ns, not significant. *P < 0.05, **P < 0.01, and ****P < 0.0001. Scale bar: 10 µm.

The fusion of lysosomes with vacuoles (phagosome or autophagosome) is the critical bactericidal mechanism of phagocytic cells (21). To analyze the fate of intracellular Cap(+) and Cap(–) strains, lysosomal-associated membrane protein 1 (LAMP-1) and acid trophic LysoTracker dye were used to track the phagolysosome and autolysosome maturation in Cap(+) and Cap(–) infected cells. The percentage of LAMP-1-positive GAS was 60.9% and 16.2% of Cap(+) and Cap(–) strains, respectively, after 4 h of infection (Fig. 2A). Additionally, acidification (evaluated by LysoTracker) gradually increased in the Cap(+) strain but not in the Cap(–) mutant (Fig. 2B). To exclude extracellular GAS, we stained extracellular GAS (before cell permeabilization) and total GAS (after cell permeabilization) with different anti-GAS antibodies after 4 h of infection (Fig. S2). The results indicated that the acidification levels were similar to those depicted in Fig. 2B. Moreover, lysosomal acidification inhibitors [chloroquine (CHQ) and bafilomycin A1 (BAFA1)] recovered Cap(+) strain growth but had no effect on Cap(–) mutant (Fig. 2C and D). These results suggested that the Cap(–) mutant would escape from acidic lysosomal clearance.

### Cap(+) and Cap(–) strains have a similar ability to rupture phagosome membrane

**Fig 3:**
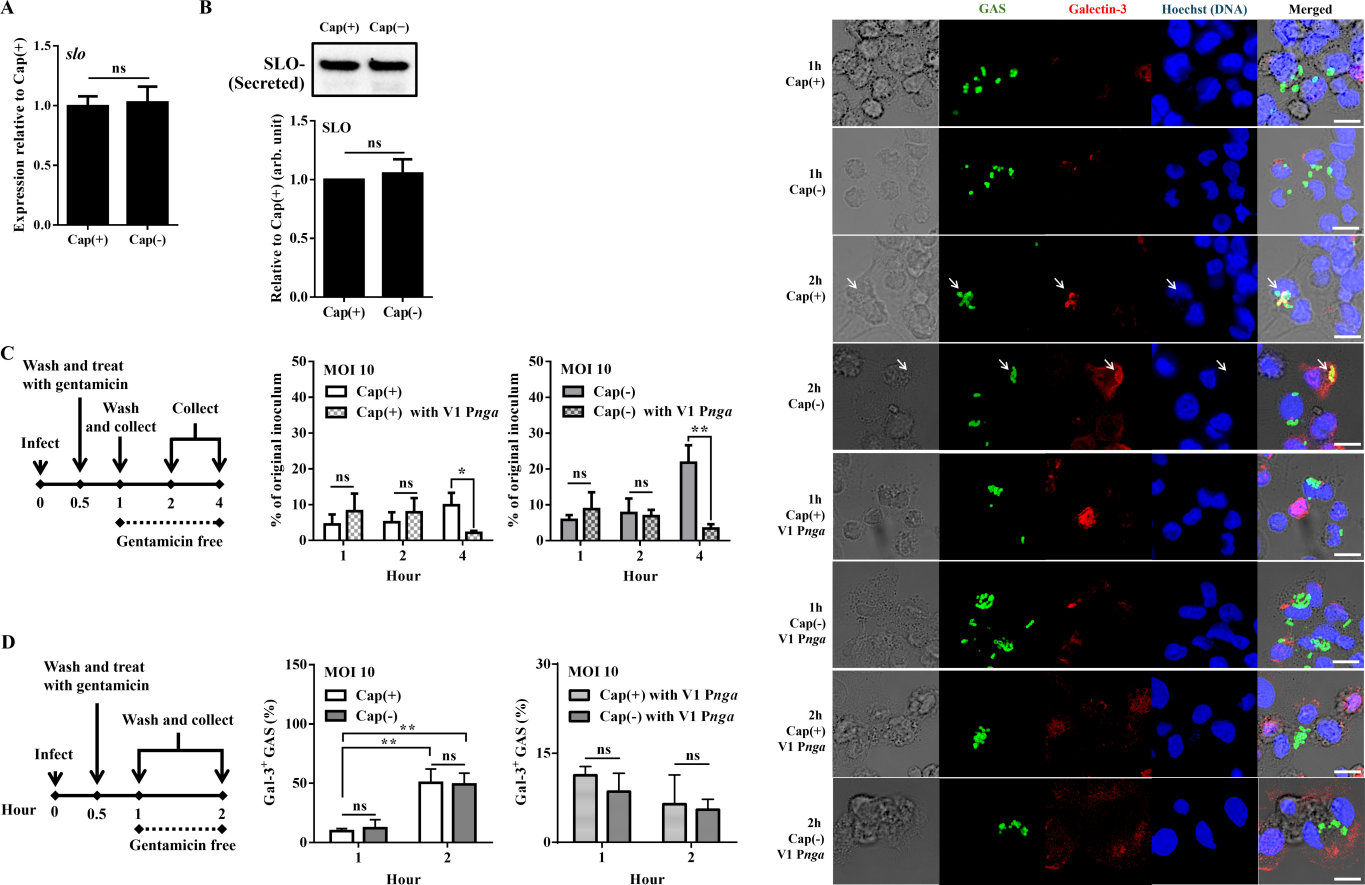
Cap(+) and Cap(–) strains have similar SLO secretion and phagosomal rupture abilities. (**A**) The expression of *slo* in the Cap(+) and Cap(–) strains in the late exponential phase of growth [the optical density at 600 nm (OD_600_) reached 1.0]. RNAs of these strains were extracted, and the expression of *slo* was analyzed by the quantitative RT-PCR. The expression of *slo* was normalized to the expression of *gyrA* for all strains. Data were analyzed by Student’s *t*-test. ns, not significant. (**B**) The amount of secreted SLO in the Cap(+) and Cap(–) strains in the late exponential growth phase. Thirty microliters of bacterial culture supernatants was analyzed by western blot with the anti-SLO antibody. Data were analyzed by Student’s *t*-test. ns, not significant. (**C**) The number of Cap(+) strain, Cap(–) mutant, and their V1 P*nga* mutants in phagocytic cells after 1–4 h of infection. GAS strains infected PMA-activated U937 cells at an MOI of 10. After incubation for 30 min, cells were incubated with gentamicin (100 µg/mL) for an additional 30 min. Afterward, the medium was replaced by the gentamicin-free medium. The infected cells and culture media were collected to determine the number of surviving bacteria by plating assay. Data were analyzed by two-way ANOVA. (**D**) The percentage of galectin-3-colocalized Cap(+) and Cap(–) strains in phagocytic cells after 1–2 h of infection. GAS strains infected PMA-activated U937 cells at an MOI of 10. After incubation for 30 min, cells were incubated with gentamicin (100 µg/mL) for an additional 30 min. Afterward, the medium was replaced by the gentamicin-free medium. Representative images of PMA-activated U937 cells infected with Cap(+) strain, Cap(–) mutant, and their V1 P*nga* mutants are shown. GAS and galectin-3 were labeled with anti-GAS antibody (green) and anti-galectin-3 antibody (red). DNA was stained with Hoechst 33342 (blue). Percentages of galectin-3-colocalized GAS were determined relative to total intracellular GAS. White arrow: galectin-3-colocalized GAS. Scale bar: 10 µm. Data were analyzed by two-way ANOVA. At least 50 infected cells were evaluated in three independent experiments (mean ± SD). ns, not significant. *P < 0.05 and **P < 0.01.

We next elucidated whether the Cap(–) mutant had a better intracellular survival activity than the Cap(+) strain. SLO, SpeB, and M proteins are virulence factors to protect GAS from intracellular killing in epithelial and phagocytic cells (11, 19, 22, 23). The transcriptional and translational expression levels of SpeB (Fig. S3A and B) and SLO (Fig. 3A and B) in the Cap(+) and Cap(–) strains were similar. In addition, the survival activity of *speB* isogenic mutants of Cap(+) and Cap(–) strains was analyzed in the macrophage infection model. The results showed the absence of SpeB reduced the survival activity of Cap(+) and Cap(−) strains after 4 h of infection in PMA-activated U937 cells (Fig. S3C). The results suggested that SpeB was required for both Cap(+) and Cap(–) strains to replicate within macrophages. Moreover, the deletion of the M protein-encoding gene (*emm*1) in the Cap(+) and Cap(–) strains did not affect the survival activity in PMA-activated U937 cells (Fig. S3C). Zhu et al. (8) showed that the high level of *slo* and *nga* expression, driven by the promoter with sequence variant 3 (P*nga*3), is required for the capsule-deficient *emm*89 strain to cause severe infection in a mouse model. In line with the study, modification of the promoter from sequence variant 3 to variant 1 (P*nga*1) reduced the expression of SLO and abolished the intracellular survival activity of Cap(+) and Cap(–) strains (Fig. 3C). These results suggested that M protein and SpeB were not critical factors to promote the intracellular survival of Cap(–) mutant. However, the Cap (–) mutant required SLO to escape into the cytosol for replication, as did the Cap (+) strain. GAS can rupture the phagosomal membrane by SLO (11, 19). With similar expression levels of SLO, Cap(+) and Cap(–) strains showed a similar percentage of galectin-3, a marker for phagosome rupture (24), during 1–2 h of infection (Fig. 3D), which was further decreased in both P*nga*1 stains. These results suggested that the Cap(+) and Cap(–) strains had similar activity to escape from phagosomes.

### Hyaluronic acid capsule is a target of selective autophagy in phagocytes

**Fig 4:**
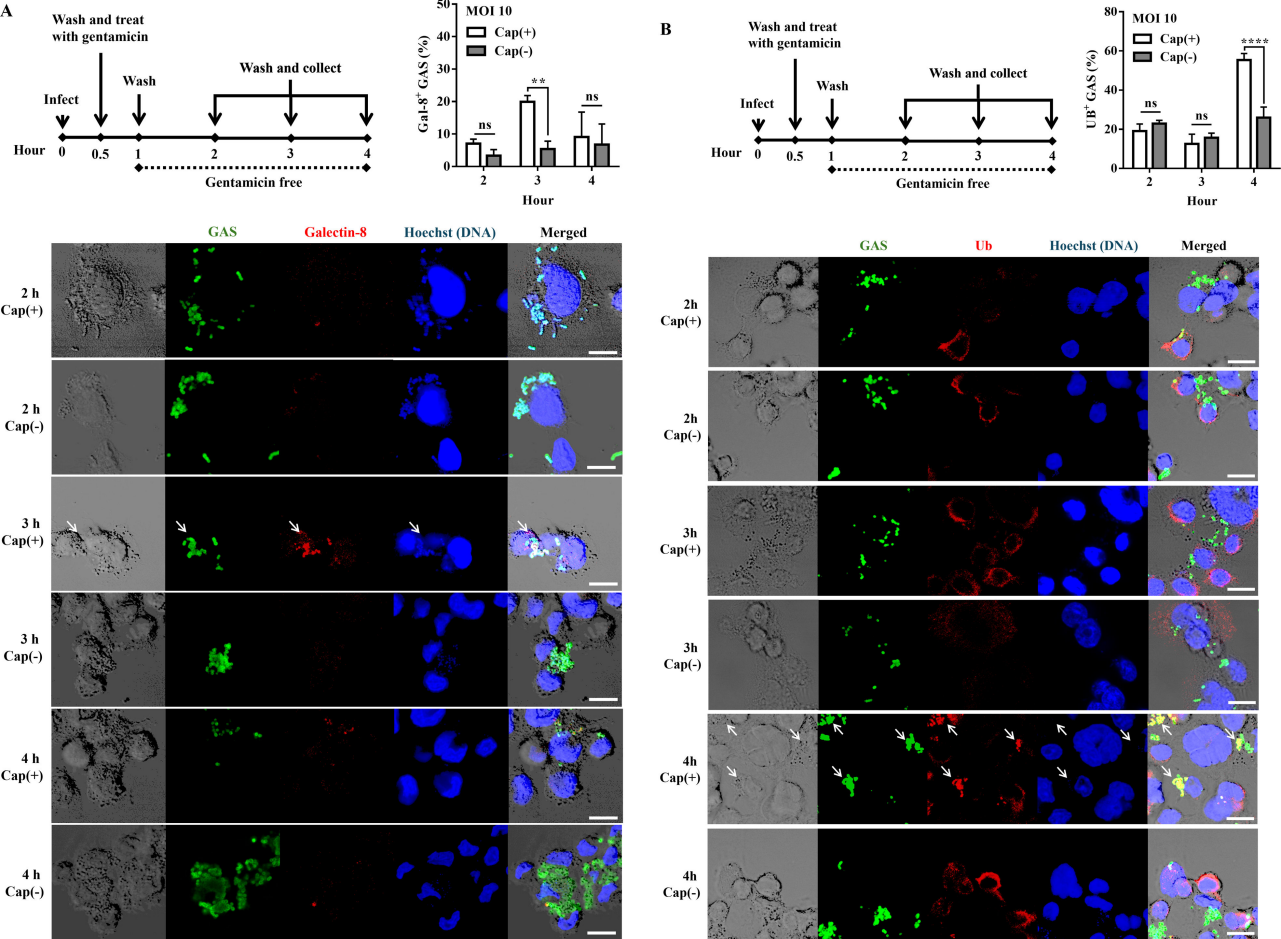
The Cap(–) mutant was ineffectively recognized by ubiquitin and galectin-8. (**A**) The percentage of galectin-8-colocalized Cap(+) and Cap(–) strains in phagocytic cells after 1–2 h of infection. GAS strains infected PMA-activated U937 cells at an MOI of 10. After incubation for 30 min, cells were incubated with gentamicin (100 µg/mL) for an additional 30 min. Afterward, the medium was replaced by the gentamicin-free medium. Representative images of PMA-activated U937 cells infected with Cap(+) and Cap(–) strains are shown. GAS and galectin-8 were labeled with anti-GAS antibody (green) and anti-galectin-8 antibody (red). White arrow: galectin-8-colocalized GAS. DNA was stained with Hoechst 33342 (blue). Data were analyzed by two-way ANOVA. (**B**) The percentage of ubiquitin-colocalized Cap(+) and Cap(–) strains in PMA-activated U937 cells after 2–4 h of infection. GAS strains infected PMA-activated U937 cells at an MOI of 10. After incubation for 30 min, cells were incubated with gentamicin (100 µg/mL) for an additional 30 min. Afterward, the medium was replaced by the gentamicin-free medium. Representative images of PMA-activated U937 cells infected with Cap(+) and Cap(–) strains are shown. GAS and ubiquitin were labeled with anti-GAS antibody (green) and anti-ubiquitin antibody (red). White arrow: ubiquitin-colocalized GAS. DNA was stained with Hoechst 33342 (blue). Percentages of galectin-8-colocalized (**A**) and ubiquitin-colocalized GAS (**B**) were determined relative to total intracellular GAS. Data were analyzed by two-way ANOVA. At least 50 infected cells were evaluated in at least two independent experiments (mean ± SD). ns, not significant. **P < 0.01 and ****P < 0.0001.

After escape from the phagosome, the autophagic isolation membrane would target the cytosolic GAS (25). For specific recognition, the cytosolic pathogens would be decorated with galectin-8 and ubiquitin, which bridge the pathogens to the isolation membrane (12, 14, 26). The colocalization of Cap(+) and Cap(–) strains with galectin-8 and ubiquitin in infected cells was detected by confocal microscope. The results showed that 19.93% of Cap(+) strain but only 5.4% of Cap(–) mutant was colocalized with galectin-8 after 3 h of infection (Fig. 4A). In addition, the results showed that 55.34% of the Cap(+) strain exhibited ubiquitination after 4 h of infection, whereas only 25.95% of the Cap(–) mutant demonstrated ubiquitination. These results suggested that the cytosolic Cap(–) mutant could escape from galectin-8 and ubiquitin-mediated recognition in the phagocytic cells.

**Fig 5:**
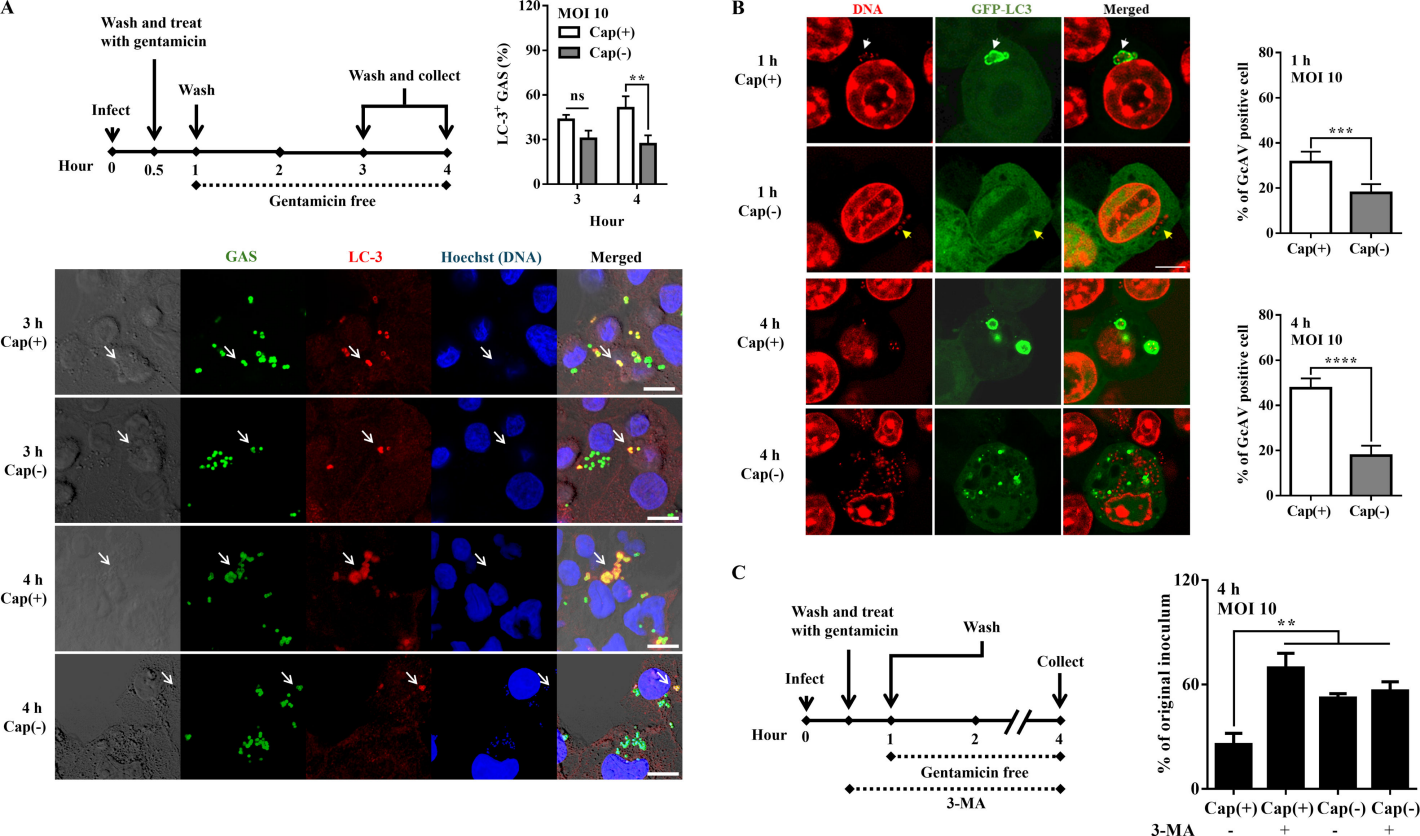
The Cap(+) strain but not the Cap(–) mutant was effectively targeted by selective autophagy. (**A**) The percentage of LC-3-colocalized Cap(+) and Cap(–) strains in phagocytic cells after 3–4 h of infection. GAS strains infected PMA-activated U937 cells at an MOI of 10. After incubation for 30 min, cells were incubated with gentamicin (100 µg/mL) for an additional 30 min. Afterward, the medium was replaced by the gentamicin-free medium. Representative images of PMA-activated U937 cells infected with Cap(+) and Cap(–) strains are shown. GAS and LC-3 were labeled with anti-GAS antibody (green) and anti-LC-3 antibody (red), respectively. White arrow: LC-3-colocalized GAS. DNA was stained with Hoechst 33342 (blue). Percentages of LC-3-colocalized GAS (**A**) were determined relative to total intracellular GAS. Data were analyzed by two-way ANOVA. At least 50 infected cells were evaluated in three independent experiments (mean ± SD). (**B**) The percentage of GAS-containing autophagosome-like vacuole)-positive phagocytic cells. Raw264.7 cells expressing GFP-LC3 were infected with GAS strains at an MOI of 10. After incubation for 30 min, cells were incubated with gentamicin (100 µg/mL) for an additional 30 min. Afterward, the medium was replaced by the gentamicin-free medium. Representative images of Raw264.7 cells infected with Cap(+) and Cap(–) strains were shown. Data were analyzed by Student’s *t*-test. Hoechst (red) was used for staining DNA, including cell nuclear and bacterial DNA. GAS was stained with Hoechst (red). White arrow: GcAV. Yellow arrow: GAS only. Scale bar: 10 µm. (**C**) The number of surviving Cap(+) and Cap(–) strains in phagocytic cells treated with the autophagy inhibitor 3-methyladenine (3-MA) after 4 h of infection. PMA-activated U937 cells were infected by GAS strains at an MOI of 10. After incubation for 30 min, cells were incubated with gentamicin (100 µg/mL) and 3-MA (10 µM) for an additional 30 min. Afterward, the medium was replaced by the gentamicin-free medium that contained 3-MA. After 4 h of infection, the infected cells and culture media were collected to determine the number of surviving bacteria by plating assay. Data were analyzed by one-way ANOVA. ns, not significant. **P < 0.01, ***P < 0.001, and ****P < 0.0001.

Next, the colocalization of Cap(+) and Cap(–) strains with LC-3 (autophagosome marker) in infected cells was detected by a confocal microscope. We employed the anti-LC3 antibody to label autophagosomes in U937 cells and Raw264.7 cells expressing GFP-LC3. In PMA-activated U937 cells, no significant difference was found in the colocalization of LC3 with Cap(+) and Cap(–) strains after 3 h of infection (Fig. 5A). After 4 h of infection, LC3 exhibited colocalization with 51.15% of the Cap(+) strain, whereas only 26.89% of the Cap(–) mutant colocalized with LC3 (Fig. 5A). In order to visualize the structure of autophagosomes, the Raw264.7 cells expressing GFP-LC3 were infected by GAS strains. We quantified the proportion of cells displaying GAS-containing autophagosome-like vacuoles (GcAV) to assess the presence of GcAV-positive cells. After 1 h of infection, 33.27% of Cap(+) infected cells displayed GcAV positivity, while only 17.05% of Cap(–) infected cells exhibited GcAV positivity (Fig. 5C).

Finally, we utilized an autophagy inhibitor 3-methyladenine (3-MA) to verify whether the selective autophagy mediates the intracellular elimination of the Cap(+) strain but not the Cap(–) mutant. Results showed that 3-MA effectively prevented the Cap(+) strain, but not the Cap(–) mutant, to be eliminated by PMA-activated U937 cells (Fig. 5B). These results suggested that the Cap(–) mutant would escape from autophagosome recognition.

## DISCUSSION

In recent years, there has been a growing body of literature highlighting the presence of capsule-deficient strains of GAS (8, 9, 27). Acapsular strains of GAS, such as the *emm89* isolate that lacks hyaluronic acid capsule production, have been shown to possess increased virulence in a mouse model of necrotizing fasciitis compared to its poorly encapsulated strain (8). This information suggests that acapsular strains possess undisclosed advantages during infection, highlighting the need for further investigation to elucidate their underlying mechanisms.

The findings of our study are highly intriguing, as the results suggest that the bacterial capsule may serve as a target in macrophages. Traditionally, it has been hypothesized that the bacterial capsule provides protection against host defense mechanisms. For example, the capsule of *Streptococcus pneumoniae* has been shown to promote bacterial intracellular survival in endothelial cells by conferring resistance to oxidative stress (28). Similarly, the capsule of *emm18* GAS contributes to nasal and skin infections by evading neutrophil-mediated clearance (29). However, contrary to these expectations, our findings demonstrate that the GAS capsule, in the global disseminated *emm1* strain, only has a minor role in providing protection against phagocytosis but supports the host’s recognition of GAS in macrophages.

The hyaluronic acid capsule of GAS has been considered as the key bacterial structure to prevent phagocytosis. For example, a recent study showed that the capsule of *emm18* GAS provides protection for neutrophil-mediated clearance (29). We evaluated the survival of *emm*1-type Cap(+) and Cap(–) strains in human whole blood and polymorphonuclear leukocytes (PMNs), and the results showed that there was no significant difference in the resistance capabilities against whole blood and PMNs between the Cap(+) and Cap(−) strains (Fig. S4A and B). Furthermore, our results demonstrated no statistically significant difference in the intracellular survival of Cap(+) and Cap(−) strains after 1 h of infection. We cannot entirely rule out a minor decrease in the phagocytosed Cap(+) strain due to the protective role of the hyaluronic acid capsule. However, this difference did not lead to increased cytotoxicity of the Cap(−) mutant (data not shown). Moreover, the Cap(−) mutant exhibited a superior ability to survive in phagocytic cells compared to the Cap(+) strain by escaping autophagy-mediated elimination. Zhu et al. (8) showed that the SLO and NADase expression driven by the P*nga*3 promoter could compensate for the loss of capsule in the *emm89* strain. Furthermore, the *emm89* acapsular strain is equally virulent to the heavily encapsulated strain but is more virulent than its poorly encapsulated mutant in a mouse necrotizing fasciitis model. These results suggest that the role of the hyaluronic acid capsule in GAS resistance to phagocytic killing would not be identical in GAS strains and could be related to both the production levels of the hyaluronic acid capsule and the activities of the *nga-ifs-slo* promoter.

**Fig 6:**
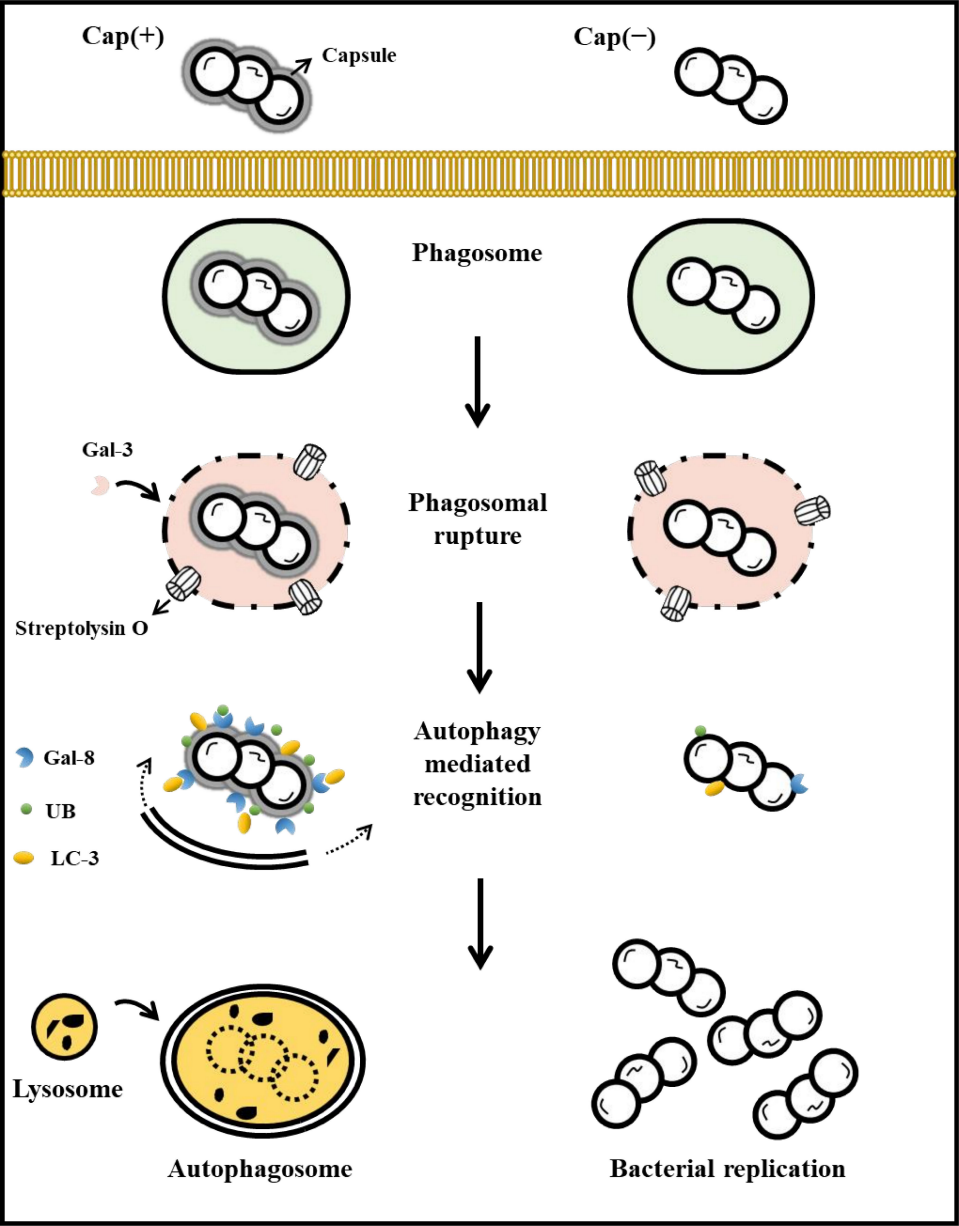
Capsule-deficient GAS can escape from selective autophagy targeting in macrophages. Schematic representation of the fate of capsulated and acapsulated GAS in macrophages. After phagocytosis, Cap(+) and Cap(–) strains have a similar ability to rupture phagosome by streptolysin O. Nonetheless, the cytosolically exposed Cap(+) strain but not Cap(–) mutant is effectively recognized by galectin-8 and ubiquitin, resulting in the formation of the autophagolysosome and intracellular killing.

During the early stages of infection, there was no significant difference between the Cap(+) and Cap(−) strains in terms of the amount of GAS that adhered to and entered cells or their ability to rupture phagosomes. However, we observed an increase in the number of the hyaluronidase-treated Cap(+) strains compared to the untreated Cap(+) strains after incubation with phagocytic cells, suggesting that the elimination of hyaluronic acid capsule would promote GAS intracellular survival in macrophages. The acidity of endosome/phagosome is pH 6.3–6.5 (30). Our previous study indicates that *hasA* expression of the Cap(+) strain is inhibited under acidic stress (pH 6.0) (31); therefore, the hyaluronidase-treated Cap(+) strain would not regenerate capsules and was not eliminated effectively after being uptaken by macrophages. After phagosomal rupture, the Cap(+) strain exhibited more effective recognition by galectin-8 and ubiquitin than the Cap(–) mutant. The previous study suggested that, upon recognition, galectin-8 could recruit and interact with the E3 ligase parkin (26) and the autophagy adapter TAX1BP1 (12) to restrict pathogen replication through selective autophagy. Therefore, the Cap(+) strain would be internalized and subsequently engaged with galectin-8, leading to the recognition of ubiquitin and activation of selective autophagy (Fig. 6). Nonetheless, the role of the interaction between the hyaluronic acid capsule and galectin-8 in autophagy activation in GAS infection needs to be further clarified.

In summary, our investigation of isogenic mutant strains and macrophage-like cells reveals that hyaluronic acid capsule deficiency in *emm1*-type GAS does not affect the function of SLO, SpeB, and M proteins in the cell infection model. Additionally, the hyaluronic acid capsule can serve as a target for autophagy-mediated killing in macrophages. The *emm1* GAS is one of the most prevalent *emm*-types worldwide (32), and GAS with strong P*nga* activity is related to increased bacterial virulence (8, 27). This study sheds light on the evolving understanding of GAS virulence and the role of capsule deficiency in its pathogenesis. Further research is needed to uncover the detailed mechanisms underlying the enhanced survival capabilities of acapsular strains and to explore their implications in the context of host-pathogen interactions and potential therapeutic strategies.

## MATERIALS AND METHODS

### Bacterial strains and growth conditions

The GAS wild-type A20 strain [Cap(+)] (*emm1*/ST28 type) is a clinical isolate described previously (33). GAS strains used in this study are listed in Table 1. GAS was grown at 37°C in tryptic soy broth (Becton, Dickinson and Company, Sparks, MD, USA) containing 0.5% yeast extract (TSBY). *Escherichia coli* strain DH5α (Yeastern Biotech Co., LTD, Taipei, Taiwan) was used for cloning. The antibiotics were used in the following concentrations: chloramphenicol, 3 µg/mL and 25 µg/mL for GAS and *E. coli*, respectively; spectinomycin, 100 µg/mL for both GAS and *E. coli*. For infection assays, gentamicin was used at 100 µg/mL.

**TABLE 1:** Bacterial strains and plasmids used in the study

	Parental strain	Description^*a*^	Reference
Strain			
Cap(+)	–*^b^*	*emm1*/ST28 wild-type strain (SCN101)	(33)
Cap(−)	Cap(+)	The *hasA* mutant (SCN156)	(20)
Cap(+) with V1 P*nga*	Cap(+)	The wild-type strain with variant V1 *nga-ifs-slo* promoter (SCN254)	This study
Cap(−) with V1 P*nga*	Cap(−)	The *hasA* mutant with variant V1 *ifs-nga-slo* promoter (SCN317)	This study
Cap(+)∆*emm*	Cap(+)	The *emm1* mutant (Cm^R^) (SCN307)	This study
Cap(−)∆*emm*	Cap(−)	The *emm1* mutant (Cm^R^) (SCN308)	This study
Cap(+)∆*speB*	Cap(+)	The *speB* mutant (Cm^R^) (SCN344)	(34)
Cap(−)∆*speB*	Cap(−)	The *speB* mutant (Cm^R^) (SCN414)	This study
Plasmid			
Vector 78	–	*cat*	(35)
pCN143	–	Temperature-sensitive vector	(36)
pCN158	–	pCN143::Δ*hasA*	(20)
pCN213	–	pCN143::Δ*emm1*Ω*cat*	This study

^*a*^ Cm, chloramphenicol; *cat,* chloramphenicol acetyltransferase.

^*b*^ –, none.

### DNA and RNA manipulations

GAS genomic DNA extraction, RNA extraction, and reverse transcription were performed as described previously (37). RNA was collected from GAS strains during the late exponential phase of growth [the optical density at 600 nm (OD_600_) reached 1.0]. Experiments involving biological replicates were conducted in duplicate, utilizing three distinct RNA preparations. The relative expression of each target gene was standardized to *gyrA* and subsequently analyzed via the ΔΔCt method, employing the 7500 software v2.0.5 (Applied Biosystems, Thermo Fisher Scientific, Inc.). Prior to statistical evaluation, all data points from both control and experimental groups were normalized to the mean of the control samples (38). The primers used for real-time PCR analysis were designed using the Primer3 v0.4.0 software (available at https://bioinfo.ut.ee/primer3-0.4.0/), in accordance with the MGAS5005 sequence (accessible via NCBI, accession no. NC_007297.1) and described in Table 2.

**TABLE 2:** Primer utilized for this study

Primer	Use	Sequence (5′−3′)^*a*^	Reference
hasA-F-2	Real-time PCR	tgaaagatctgacgctgacg	(31)
hasA-R-2	Real-time PCR	accccaaaggcattatcgta	(31)
slo-F-1	Real-time PCR	gcagagcacaataaggtagt	(31)
slo-R-1	Real-time PCR	ctggtgtatgaaataggataag	(31)
speB-2-F	Real-time PCR	tgcctacaacagcactttgg	(36)
speB-2-R	Real-time PCR	ggtaaagtaggcggacatgc	(36)
gyrA-F-3	Real-time PCR	cgtcgtttgactggtttgg	(36)
gyrA-R-3	Real-time PCR	ggcgtgggttagcgtattta	(36)
emm1-F-4	Construction	gcgggatccacgcctttttcatcacttgg	This study
emm1-R-4	Construction	gcgggatcctcttcaggccaatcttcagg	This study
Inv-emm1-SacII-F	Construction	tccccgcggtaagctatcactttgtaatac	This study
Inv-emm1-SacII-R	Construction	tccccgcggcatttttatgctccttatgctat	This study
pnga-F-1	Construction	gcgggatcccgaagaaaatcgctttccag	This study
pnga-R-1	Construction	gcgggatccaagatcgcgttccatattgc	This study

^*a*^ Underline indicates restriction enzyme site.

### Cell culture and infection model

The human histiocytic lymphoma cell line U937 and THP-1 cells were maintained at 1 × 10^6^ cell/mL concentration in RPMI medium supplemented with 10% fetal bovine serum (FBS) (Gibco, Invitrogen, Carlsbad, CA, USA) at 37°C and 5% CO_2_ conditions. U937 and THP-1 cells were differentiated into macrophages before infection by stimulating with 15 nM of phorbol myristate acetate for 3 days. The mouse macrophage cell line RAW264.7 cells were maintained in DMEM medium (Sigma-Aldrich, D6429) with 10% FBS and infected with GAS directly without pre-stimulation.

The overnight bacterial cultures were transferred to fresh TSBY broth at a 1:50 dilution and grew to an optical density at 600 nm of 0.5–0.6. The bacterial cultures were washed with 1× PBS and resuspended in an FBS-free medium. Before infection, the cells were treated with or without cytochalasin D (10 mg/mL). PMA-activated U937 cells were infected with GAS at an MOI of 10 in an FBS-free medium. Plates were centrifuged at 335 *× g* for 3 min to facilitate and synchronize infection. After 30 min of infection, the infected cells were washed and treated with gentamicin (100 µg/mL), chloroquine (50 µM), bafilomycin A1 (100 nM), or 3-methyladenine (10 mM) depending on different experimental conditions. After infection, cells were washed with 1× PBS twice and lysed with 0.1% Triton X-100 for 5 min at room temperature. The cell lysates or culture medium supernatant were serially diluted with 1× PBS. The number of surviving GAS was evaluated by the plating method.

### Immunofluorescence microscopy

PMA-activated U937 cells or Raw264.7 cells were seeded onto glass coverslips in 6-well plates and infected with GAS. After infection, coverslips were washed and fixed by 4% paraformaldehyde in 1× PBS for 10 min, permeabilized by 1× PBS containing 0.1% Triton X-100 for 10 min, and incubated with 0.2% gelatin in 1× PBS for 1 h. The cells inoculated with primary antibodies (anti-GAS, PAB13831, and PAB29815, Abnova; anti-galectin-3, sc-23938, Santa Cruz; anti-galectin-8, sc-377133, Santa Cruz; anti-ubiquitin ENZ-ABS840, Enzo; anti-LAMP-1, MABC1108, Merck) were prepared in 0.2% gelatin in 1× PBS at 4°C overnight. Cells were washed twice with 1× PBS and incubated with Alexa Fluor-conjugated secondary antibodies (111-545-003, 211-605-109, and 705-165-003, Jackson Immunoresearch) with or without Hoechst (10 µg/mL) containing 0.2% gelatin at room temperature for 1 h. Cells were washed twice in 1× PBS and mounted onto a microscope slide. Fixed cells were imaged with a laser-scanning microscope (LSM780, Zeiss). For LysoTracker labeling, infected cells were incubated with a 40 nM LysoTracker (L7528, Thermo Fisher Scientific) probe for 30 min before fixation.

### Western blot

After SDS-PAGE electrophoresis, the separated protein was transferred to the polyvinylidene fluoride (PVDF) membrane. The PVDF membrane was incubated with 5% skim milk in PBST buffer (1× PBS containing 0.2% of Tween-20) at room temperature for 1 h. After incubation, the membrane was hybridized with the primary antibodies (anti-SpeB, PBI222, Toxin Technology; anti-SLO, GTX64717, GeneTex) with appropriate dilution at 4°C for 12–16 h. After hybridization, the membrane was washed three times with PBST. The membrane was hybridized with a secondary antibody (anti-mouse or anti-rabbit IgG-HRP) with 1:10,000 dilution at room temperature for 1 h. The blot was developed using Pierce ECL western Blotting Substrate (Thermo Fisher Scientific Inc., Rockford, IL, USA), and a signal was detected by the Gel Doc XR + system (BioRad, Hercules, CA, USA).

### Statistics

Statistical analysis was performed by Prism software (GraphPad, version 6.01). The data were expressed as mean ± SD. *P* values < 0.05 were considered statistically significant. Significant differences were calculated by Student’s *t*-test, one-way analysis of variance with Tukey’s multiple comparisons test, and two-way ANOVA with Sidak’s multiple comparisons test.

## SUPPLEMENTAL MATERIAL

Supplemental figures mbio.00771-24-s0001.docx Figures S1 to S4.

ASM does not own the copyrights to Supplemental Material that may be linked to, or accessed through, an article. The authors have granted ASM a non-exclusive, world-wide license to publish the Supplemental Material files. Please contact the corresponding author directly for reuse.
