# Supplementary material for: Capsule-deficient group A Streptococcus evades autophagy-mediated killing in macrophages
Source: mBio. 2024 May 31;15(7):e00771-24. doi: 10.1128/mbio.00771-24 (PMC11253618; doi:10.1128/mbio.00771-24)
Supplement: Supplemental figures — Figures S1 to S4. [file mbio.00771-24-s0001.docx]

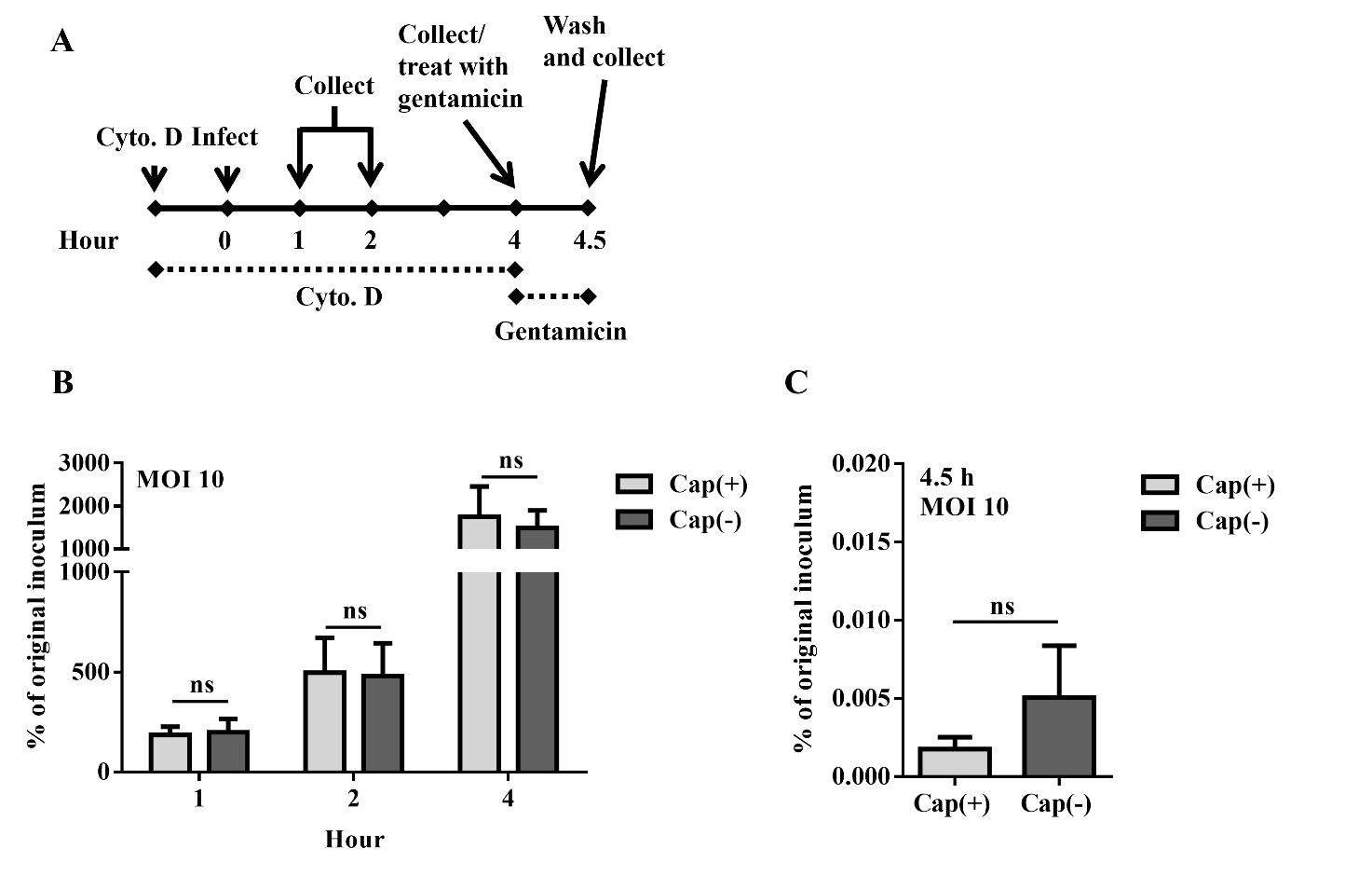


**Figure S1. The Cap(+) and Cap(–) strains had a similar activity to replicate in the extracellular niche of phagocytic cells. (A)** The extracellular replication activity and number of intracellular Cap(+) and Cap(–) strains after 1–4.5 h of infection. The PMA-activated U937 cells were treated with cytochalasin D (10 mg/mL) for 1 h before infection. GAS strains infected the cells at MOI 10 for 4 h. **(B)** The infected cells and culture media were collected to determine the number of surviving bacteria by the plating assay. Data were analyzed by two-way ANOVA. **(C)** The infected cells were treated with gentamicin (100 μg/mL) for 30 min, and the infected cells were collected to determine the number of intracellular bacteria. Data were analyzed by Student *t*-test. ns, not significant. The mean ± SD of three independent experiments is shown.


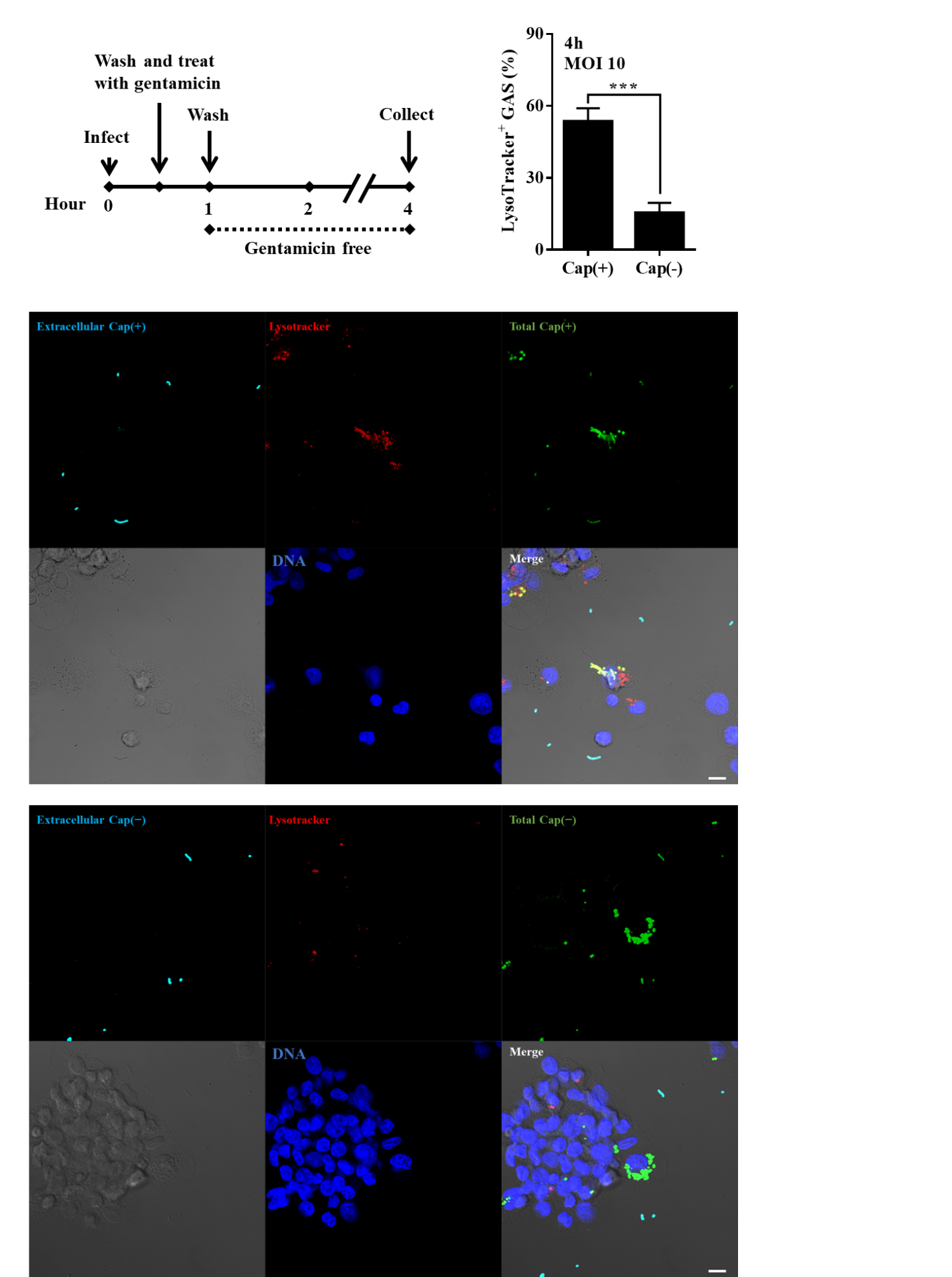


**Figure S2. The level of lysosome colocalization with intracellular Cap(+) and Cap(−) strains.** The level of lysosome-associated Cap(+) and Cap(–) strains in PMA-activated U937 cells. GAS strains infected PMA-activated U937 cells at MOI 10. After incubation for 30 min, cells were incubated with gentamicin (100 µg/mL) for an additional 30 min. Afterward, the medium was replaced by the gentamicin-free medium. Representative images of PMA-activated U937 cells infected with Cap(+) strains and Cap(–) mutants by confocal microscopy were shown. GAS-infected U937 cells were stained with LysoTracker dye (Red) for 30 min prior to each time point. Before the cells were permeabilized, extracellular GAS was detected by rabbit anti-GAS antibodies (cyan). After permeabilization, goat anti-GAS antibodies were used for detection (green, scale bar: 10 μm). DNA was stained with Hoechst 33342 (blue). The results showed that the percentage of Lysotracker-positive Cap(+) and Cap(−) strains was 53.6% and 15.4%, respectively. Data were analyzed by two-way ANOVA. LysoTracker-positive GAS was determined relative to total intracellular GAS. At least 50 infected cells were evaluated in three independent experiments (mean ± SD).


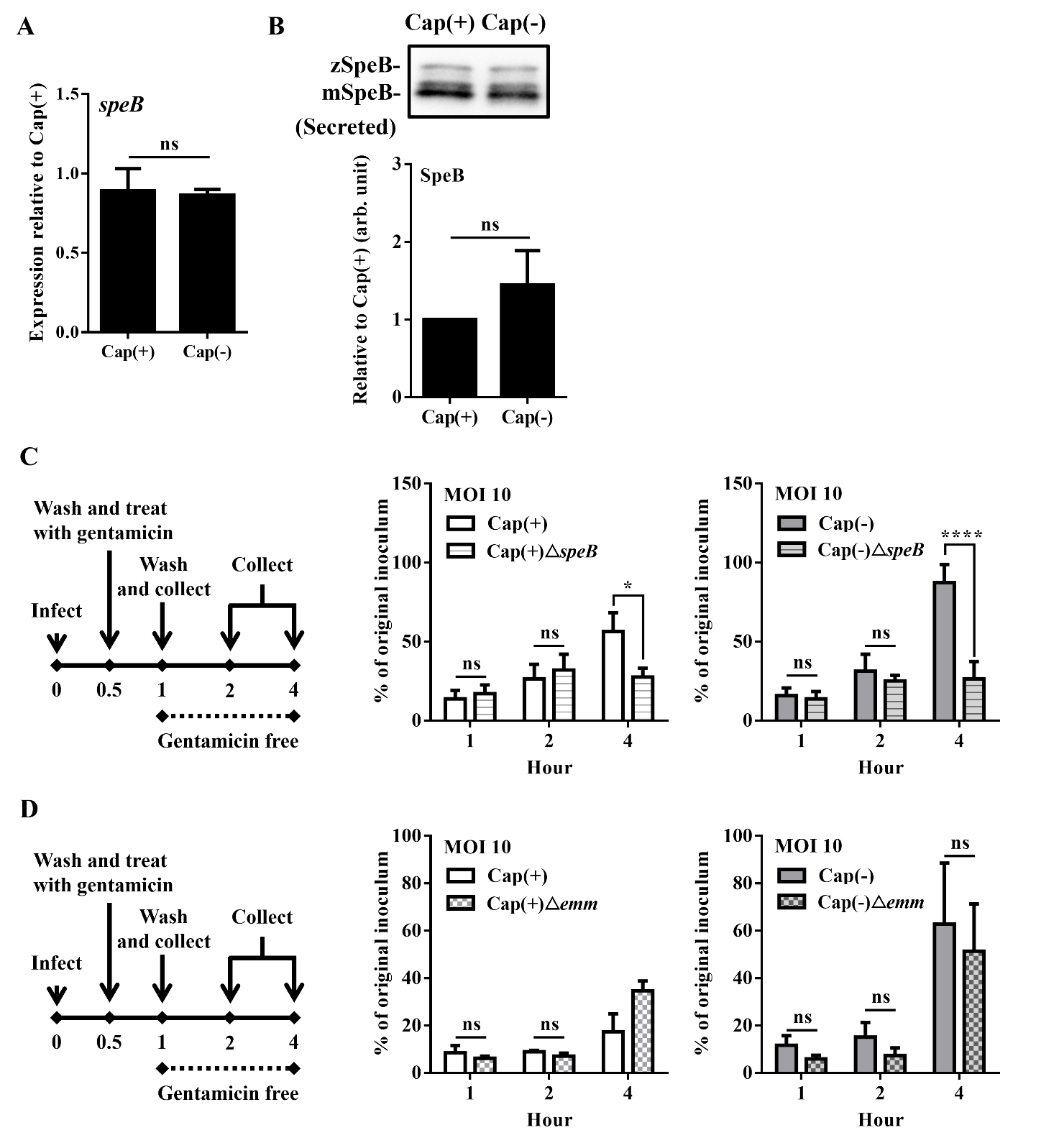


**Figure S3. SpeB and M protein were not critical factors to promote the intracellular survival of Cap(–) mutant. (A)** The expression of *speB* in Cap(+) and Cap(–) strains in the late exponential phase of growth (the optical density at 600 nm [OD600] reached 1.0). RNAs of these strains were extracted, and the expression of *speB* was analyzed by the quantitative RT-PCR (RT-qPCR). The expression of *speB* was normalized to the expression of *gyrA* for all strains. Data were analyzed by Student *t*-test. ns, not significant. **(B)** The amount of secreted SpeB in the Cap(+) and Cap(–) strains in the late exponential growth phase. Thirty-μl of bacterial culture supernatants were analyzed by western blot with the anti-SpeB antibody. Data were analyzed by Student *t*-test. ns, not significant. zSpeB, zymogen form SpeB; mSpeB, mature form SpeB. **(C)** and **(D)** The number of Cap(+) strain, Cap(–) mutant, and their *speB*/*emm1* mutants (Δ*speB*/Δ*emm*) in macrophages after 1-4 h of infection. GAS strains infected PMA-activated U937 cells at MOI 10. After incubation for 30 min, cells were incubated with gentamicin (100 μg/mL) for an additional 30 min. Afterward, the medium was replaced by the gentamicin-free medium. The infected cells and culture media were collected to determine the survival bacteria by plating assay. Data were analyzed by two-way ANOVA. ns, not significant. *, *P* < 0.05, ****, *P* < 0.0001The mean ± SD of three independent experiments is shown.


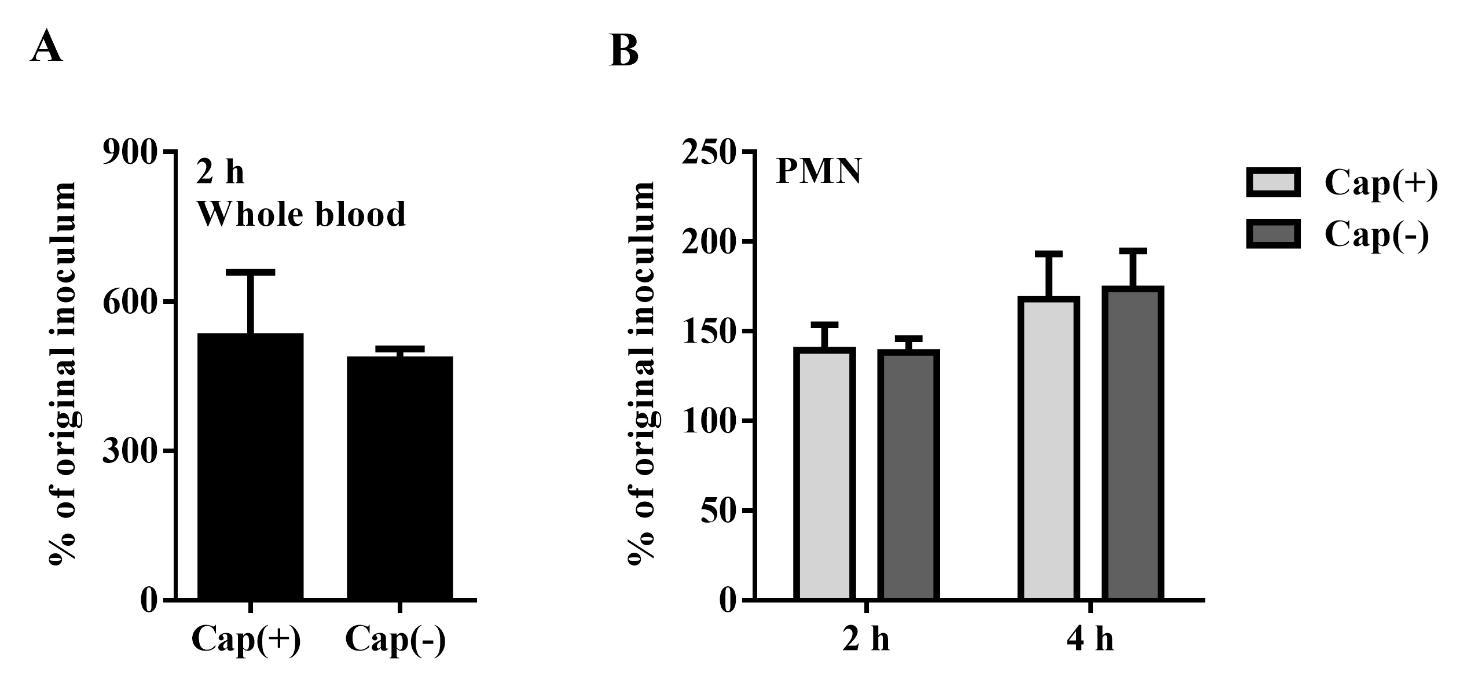


**Figure S4. Survival of Cap(+) and Cap****(–) strains in human whole blood and** **polymorphonuclear leukocytes. (A)** The number of Cap(+) and Cap(–) strains in human whole blood. GAS strains were incubated with heparinized human whole blood for 2 h. The number of surviving GAS was determined by plating assay. Data were analyzed by Student *t*-test. ns, not significant. **(B)** The number of Cap(+) and Cap(–) strains in human polymorphonuclear leukocytes (PMNs). The PMNs were infected by GAS strains at MOI 10 for 2 h and 4 h. The infected cells and culture media were collected to determine the number of surviving bacteria. Data were analyzed by two-way ANOVA. ns, not significant.
